# Effect of ZrO_2_ paste, surface treatments, and storage on Weibull characteristics and resin bond strength to zirconia

**DOI:** 10.1590/0103-6440202405581

**Published:** 2024-03-22

**Authors:** Anne Heloyse Teixeira Crispim, Sarah Emille Gomes da Silva, Fernanda Gurgel de Gois Moreira, Bianca Cristina Dantas da Silva, Amanda Maria de Oliveira Dal Piva, Liliana Gressler May, Natália de Freitas Daudt, Rodrigo Othávio Assunção Souza

**Affiliations:** 1 Federal University of Rio Grande do Norte(UFRN), Department of Dentistry, Natal, Brazil.; 2 Academic Centre for Dentistry Amsterdam (ACTA), Universiteit van Amsterdam and Vrije Universiteit, Department of Dental Materials Science, Gustav Mahlerlaan, LA Amsterdam.; 3 Dept of Restorative Dentistry, Federal University of Santa Maria, RS, Brazil.; 4 Department of Mechanical Engineering, Federal University of Santa Maria, Santa Maria, RS Brazil.

**Keywords:** Ceramics, Zirconium, Shear Strength

## Abstract

The objective is to evaluate the effect of different surface treatments and storage on the shear strength of ultratranslucent zirconia. 36 blocks of ultra-translucent zirconia were fabricated (7x7x2mm) and sintered. Then, divided into 12 groups according to the “surface treatment” (C -Primer; Al -Sandblasting with Al2O3 + Primer; Si -Silicate + Primer; Gl -Glaze + HF + Primer; Z -Zirlink; Zp -Zirlink + Primer) and “storage” factors (ST-with 150 days/37º and without). After surface treatment, five cylinders (Ø=2mm; h=2.0mm) of resin cement (n=15) were constructed in each ceramic block; at the end, the shear strength test was performed (1mm/min, 50Kgf), and analysis of surface failures. 60 additional samples (2x2x2mm) were made for extras analysis (surface roughness, MEV, and EDS). Bond strength and surface roughness data were statistically evaluated by ANOVA (2 factors/1 factor), Tukey test (5%), and Weibull analysis, respectively. ANOVA (2-way) revealed that all factors were statistically significant for bond strength. The silicatization groups (Si_ST_: 30.47^A^MPa; Si: 29.21^A^MPa) showed the highest bond strength values, regardless of storage (Tukey's test). While the groups treated with Zirlink (Z_ST_: 2.76^F^MPa; Z: 5.27^EF^MPa) showed the lowest values, just similar to the Gl_ST_ group (5.14^EF^MPa). The Weibull modulus (m) showed a statistical difference between groups (p=0.000). ANOVA (1 factor) revealed that the "surface treatment" factor (p=0.0000) was statistically significant for surface roughness. Therefore, the application of Zirlink and Glaze on pre-sintered zirconia did not promote efficient adhesion of the ultratranslucent zirconia to the resin cement, even when associated with a primer containing MDP.

## Resumo

O objetivo é avaliar o efeito de diferentes tratamentos superficiais e do envelhecimento na resistência ao cisalhamento da zircônia ultratranslúcida. Foram confeccionados 36 blocos de zircônia ultratranslúcida (7x7x2mm) e sinterizados. Em seguida, divididos em 12 grupos de acordo com o “tratamento de superfície” (C-Primer; Al-Jateamento com Al2O3+Primer; Si-Silicato+Primer; Gl -Glaze+HF+Primer; Z-Zirlink; Zp-Zirlink+Primer) e fatores de “armazenamento” (ST-com, 150 dias/37º e sem). Após o tratamento superficial, foram construídos cinco cilindros (Ø=2mm; h=2,0mm) de cimento resinoso (n=15) em cada bloco cerâmico; ao final foi realizado o ensaio de resistência ao cisalhamento (1mm/min, 50Kgf) e análise de falhas superficiais. Foram confeccionadas 60 amostras adicionais (2x2x2mm) para análises extras (rugosidade superficial, MEV e EDS). Os dados de resistência de união e rugosidade superficial foram avaliados estatisticamente por ANOVA (2 fatores/1fator), teste de Tukey (5%) e análise de Weibull, respectivamente. ANOVA (2 fatores) revelou que todos os fatores foram estatisticamente significativos para a resistência de união. Os grupos de silicatização (Si_ST_: 30,47^A^MPa; Si: 29,21^A^MPa) apresentaram os maiores valores de resistência de união, independente do armazenamento (Tukey). Enquanto os grupos tratados com Zirlink (Z_ST_: 2,76^F^MPa; Z: 5,27^EF^MPa) apresentaram os valores mais baixos, apenas semelhantes ao grupo Gl_ST_ (5,14^EF^MPa). O módulo de Weibull (m) apresentou diferença estatística entre os grupos (p=0,000). A ANOVA (1 fator) revelou que o fator “tratamento superficial” (p=0,0000) foi estatisticamente significativo para rugosidade superficial. Portanto, a aplicação de Zirlink e do Glaze na zircônia pré-sinterizada não promoveu adesão eficiente da zircônia ultratranslúcida ao cimento resinoso, mesmo quando associada a primer contendo MDP.

## Introduction

Zirconia is a polycrystalline material that has high hardness, biocompatibility, wear resistance, and bending resistance ^1^. Advances in the microstructure of this ceramic enabled development of three generations of zirconia: conventional (first generation), translucent (second generation), and ultratranslucent (third generation) ^2^. Such improvements were due to the interest of the dental market in monolithic ceramics with better aesthetics, considering that it was initially frequent to use glass-ceramic coverings with better translucency and aesthetic properties in zirconia copings due to the opaquer characteristic of conventional zirconia; however, such a procedure was subject to high levels of wear, cohesive failures and chipping ^3^. In this regard, ultra-translucent zirconia has gained great prominence in modern dentistry thanks to its ability to add excellent aesthetic properties, in addition to mechanical properties, thus becoming a truly versatile ceramic with greater clinical applications, as well as for aesthetic areas due to the use of ceramic laminates ^4^.

Despite the limited amount of available studies, the literature reports a survival rate for single crowns and monolithic fixed dental prostheses in zirconia of 94.2% and 95.7% respectively, after an average of 5.7 years of follow-up ^5^. However, despite this high success rate, previous studies address that several factors can influence the clinical longevity of zirconia restorations, including the surface treatment of zirconia before cementation ^6^. Considering the low vitreous content of zirconia ceramics, conventional etching methods with hydrofluoric acid and silane application on the surface are not capable of effectively changing the topography of these pieces and favoring their adhesion ^7^. Therefore, several protocols have been suggested for the surface treatment of zirconia, such as sandblasting with aluminum oxide particles (Al_2_O_3_) or aluminum oxide coated with silica (SiO_2_) ^6^. However, some studies have reported that the metastable nature of tetragonal zirconia can be affected by excessive abrasion with Al_2_O_3_, potentially resulting in cracking and a subsequent decrease in mechanical strength. ^1^ ^,^ ^8^.

In this context, addressing this limitation has prompted the exploration of alternative protocols, as proposed in the literature, such as laser surface treatments, that aim to enhance surface roughness, wettability, and bonding strength between zirconia and resin cement. Despite the improvements achieved, they have not surpassed the resistance values to shear obtained through abrasion by air particles ^3^ ^,^ ^9^. However, a recent development is the ZrO2 suspension (Zirlink, Blue Dent Dental, Pirassununga, Brazil), which, according to the manufacturer, is capable of promoting seven times greater adhesion to zirconia compared to blasting with Al_2_O_3_. Also, according to the manufacturer, this process is due to the presence of yttria-compatible nanoceramic components, which ensures the formation of an adhesion layer on the inner surface of the ceramic even before sintering, allowing better adhesion to the cement.

There are a quite few research studies on the use of this new ZrO_2_ suspension protocol as a surface treatment for ultratranslucent zirconia and its effectiveness compared to conventional techniques. Therefore, the objective of the present study was to evaluate the effect of different surface treatment protocols on the shear bond strength of ultratranslucent zirconia. The tested hypotheses were: 1) Zirlink presents higher shear bond strength values compared to other surface treatment methods for ultratranslucent zirconia, and 2) different surface treatment protocols affect the surface roughness of ultratranslucent zirconia.

## Materials and methods

**Figure 1:**
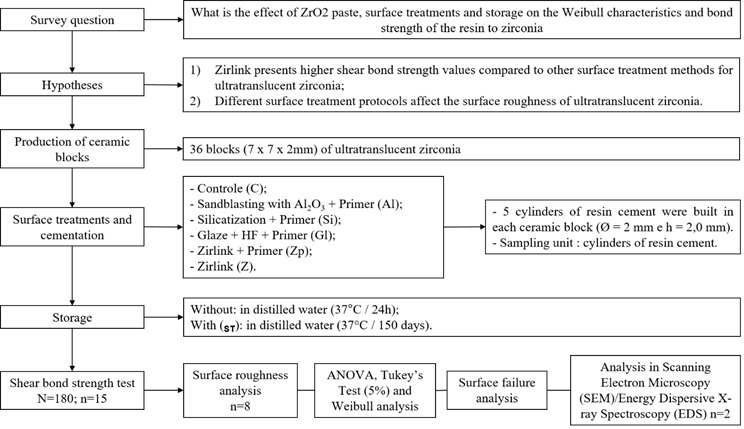
Flowchart of the research design.

The materials used in the present study, as well as the respective trademarks, are shown in Box 1. The research design is shown in Figure. 1.

**Box 1:**
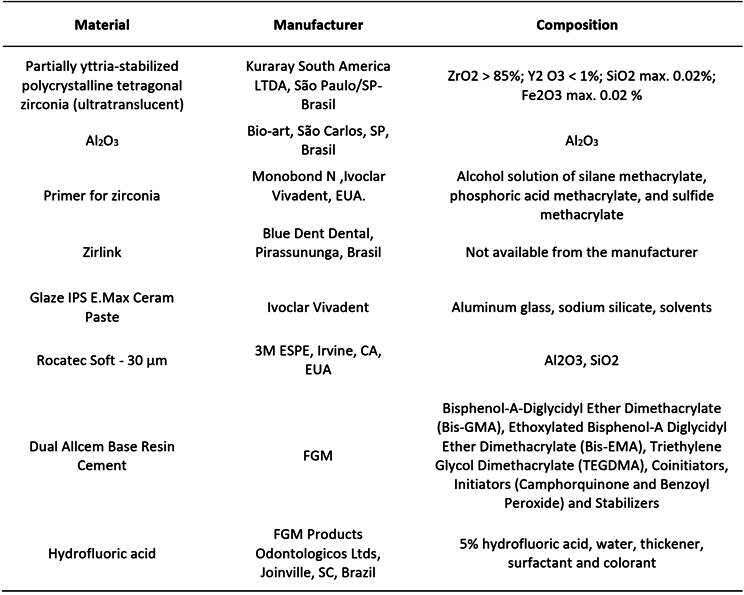
List of materials used in the study. Trade name, material, composition, and manufacturer.

### Bond strength

### Production of ceramic blocks

Ultratranslucent zirconia discs (Katana Zirconia Block, Kuraray South America LTDA, São Paulo, Brazil) measuring 15.2 x 15.2 x 38 mm were sectioned using a universal cutting machine (Odeme Dental Research, Luzerna, SC, Brazil) to obtain 36 smaller blocks (9 x 9 x 3mm). The dimensions of the blocks were verified with a digital caliper (Eccofer, Curitiba/Paraná, Brazil) after their surfaces were smoothed with abrasive water sandpaper with increasing granulation (# 600, # 800, and # 1200, 3M ESPE/Irvine, CA, USA).

Then, the blocks were cleaned in an ultrasonic device (Cristófoli Equipamentos de Biossegurança LTDA, Paraná, Brazil) for 5 min with distilled water, dried, and subsequently sintered according to the protocol recommended by the manufacturer: temperature of 1550℃ (2822°F) with a heating rate of 10℃/min. (18°F/min.), and cooling rate of -10℃/min (-18°F/min.) in a 2-hour sintering process. Considering that the ceramic sintering shrinkage is approximately 20%, the final dimensions of the blocks were 7 x 7 x 2 mm, verified using a digital caliper (Eccofer, Brazil).

The blocks were then randomly divided into 12 groups according to the “storage” (with 150 days/240℃ and without) and “surface treatment” (Control; Sandblasting with Al_2_O_3_ + primer; Silicate + primer; Glaze + HF + primer; Zirlink + primer and Zirlink) factors. The blocks in the glaze-treated groups received the application of a single thin layer of glaze, on the block’s surface using a flexible metal spatula, followed by firing the material in an oven (450^0^ - 850℃/70℃ per minute), as recommended by the manufacturer. The glaze layer was applied by the same operator. The Zirlink blocks were prepared according to the manufacturer: the flask was shaken for 5 seconds and the material was applied with a brush and the aid of a pipette on the block’s surface to receive cementation before sintering, to create the promised adhesion layer.

Afterward, the zirconia blocks were embedded in chemically activated acrylic resin (JET, Artigos Odontologistas Clássico, Brazil) with an industrial silicone mold for duplication (silicone Master- Talmax/Brazil) so that the surfaces to receive the respective surface treatments were exposed on one face of the acrylic resin block. To prevent resin from flowing onto the treated surface, the blocks were only included when the acrylic resin was in the rubbery phase, in this way, the blocks remained stable on the surface of the resin. Additionally, any blocks in which resin leaked to the surface were excluded from the study, and new blocks were made. The remaining groups were then sanded in a Polishing machine (Labpol 8-12, Extec, USA) under water irrigation with water sandpaper (#600, #800, #1200 - 3M, St.Paul, USA) to remove excess acrylic resin until a smooth and polished zirconia surface is obtained, again exposed on the resin block. In the end, 36 blocks of acrylic resin were obtained with the zirconia included in the center of the block.

### Surface treatments and cementation

Next, the ceramic surfaces were treated according to the experimental groups (N=180, n=15):

- Control: Application of the zirconia primer (Monobond N, Ivoclar Vivadent, USA) with a microbrush for 20 s, followed by light jets of air for 5 s to evaporate the solvent.
- Sandblasting with Al_2_O_3_ + Primer: The surface of the ceramic blocks was blasted with 50µm aluminum oxide particles (20 seconds, 2.5 bar, 90° inclination, 10 mm distance) using an adapted microblaster (Microjet Standard Bioart, São Carlos, SP, Brazil). The surfaces were subsequently cleaned in an ultrasonic tank with distilled water for 2 minutes and dried with an air jet. The primer for zirconia Monobond N (Ivoclar Vivadent, USA) was applied, as previously described.
- Silicatization + Primer: Sandblasting with the Rocatec System (3M/ESPE) - aluminum oxide coated with silica 30µm - silicatization, occurred in the same way as described for sandblasting with aluminum oxide. The primer for zirconia Monobond N (Ivoclar Vivadent, USA) was applied, as previously described.
- Glaze + HF + Primer: As previously described, a single thin layer of glaze was applied to the block's surface. The samples were conditioned with 5% hydrofluoric acid (Condac Porcelana, FGM, Brazil) for 20s, washed with jets of water for the same time, and dried for 30s. The primer for zirconia Monobond N (Ivoclar Vivadent, USA) was applied, as previously described.
- Zirlink + Primer: The ceramic block surfaces were treated with Zirlink (Blue Dent Dental, Pirassununga, Brazil), as previously described for the inclusion of the blocks in the acrylic resin molds. The primer for zirconia Monobond N (Ivoclar Vivadent, USA) was applied, as previously described.
- Zirlink: Block surfaces were only treated with Zirlink (Blue Dent Dental, Pirassununga, Brazil) before sintering, as previously described.

After the treatments, resin cement cylinders (Allcem, FGM, ESPE, Brazil) were built on the ceramic cementation surface in all experimental groups. A Teflon matrix (Ø = 2 mm and h = 2.0 mm) (Ultradent Jig, Ultradent, South Jordan, UT, USA) was used to standardize the adhesive area diameter and the cement increment height. The matrix center coincided with the cementation face center of the ceramic so that the entire cement layer was in contact with the ceramic.

After the adaptation of the matrix, the cement was dispensed in the matrix center and light cured for 40s (1200 mW/cm^2^ - Radii Cal, SDI, Australia). It took 12 hours for the chemical polymerization of the cement to be completed, and then the silicone matrices were removed with a scalpel blade (no.12), coupled to a scalpel handle no.3. Finally, five cylinders of resin cement were built in each ceramic block ^10^, totaling 180 samples, with 30 samples for each surface treatment.

### Storage

Half of the samples (n=15) of each surface treatment were subjected to storage by storage in distilled water in an incubator (HeraCell 150, Heraeus, Hanau, Germany) at 37°C for 150 days ^11^. The other half (n=15) was stored in distilled water at 37°C (24h), and subjected to the shear bond strength test.

### Shear bond strength test

For the shear bond strength test, the blocks were fixed with a metallic device to a universal testing machine (INSTRON 3365, Norwood, USA) so that the resin cement/ceramic interface was perpendicular to the horizontal plane (ISO 11405/2003). A chisel-shaped device (Odeme Biotechnology/Brazil) coupled to the universal testing machine with a 50 Kgf load cell applied the load to the ceramic/resin cement interface at a constant speed of 1mm/min until the specimen failed.

The calculation of bond strength was performed using the formula: R=F/A, where R= bond strength (MPa); F= force (N); A=interfacial area (mm). The adhesive area of each ceramic block was defined by the area of a circle, calculated by the following formula: A=πr^2^, where π = 3.14 and r = 1 mm, in which the radius (r) corresponds to half the diameter of the cylinder.

### Surface roughness analysis

Ultratranslucent zirconia discs (KATANA Zirconia Block, Kuraray South America LTDA, São Paulo, Brazil) measuring 15.2 x 15.2 x 38 mm were sectioned using a universal cutting machine (Odeme Dental Research, Luzerna, SC, Brazil) to obtain 48 smaller squares (3 x 3 x 3mm). The dimensions of the blocks were verified with a digital caliper (Eccofer, Curitiba/Paraná, Brazil) after their surfaces were smoothed with abrasive water sandpaper with increasing granulation (# 600, # 800, and # 1200, 3M ESPE / Irvine, CA, USA).

Then, the blocks were cleaned in an ultrasonic device (Cristófoli Equipamentos de Biossegurança LTDA, Paraná, Brazil) for 5 min with distilled water, dried, and subsequently sintered according to the protocol recommended by the manufacturer, as described above. Considering that the ceramic sintering shrinkage is approximately 20%, the final dimensions of the blocks were 2 x 2 x 2 mm, verified using a digital caliper (Eccofer, Brazil).

Then, the blocks were randomly divided into 6 experimental groups (n=8), in which the studied surface treatments were applied, as previously described. The “storage” factor was not included in this analysis. The samples were subjected to qualitative analysis of three-dimensional geometry (3D) and surface roughness measurement (mean roughness: Ra; mean square roughness: Rq; and mean roughness in the Z dimension: Rz). To do so, each sample was fixed with a double-sided tape on a test table and the roughness test was performed on the surfaces using a digital roughness meter (Mitutoyo SJ 210, Japan). Surface roughness values were obtained in µm.

### Surface failure analysis

All specimens tested for shear strength were analyzed in a stereomicroscope (Nikon SMZ800) to classify the types of failures in a) adhesive - between the ceramic/cement interface; b) mixed 1 - adhesive between the cement/ceramic + cohesive ceramic interface; c) mixed 2 - adhesive between the cement/ceramic + cement cohesive interface; and d) ceramic cohesive. Some samples from each experimental group were selected and analyzed by SEM (Bruker).

2.4 Analysis in Scanning Electron Microscopy (SEM)/Energy Dispersive X-ray Spectroscopy (EDS)

First, two extra samples were made for each experimental group for the analysis of the topographical changes resulting from the different surface treatments on the ceramic, exactly as previously described for the surface roughness analysis samples (the same samples were considered for the two Zirlink groups). These ceramic blocks were metalized with gold particles (BAL-TEC SCD 005) for 130 seconds at a current of 15mA to obtain a layer 80 Å thick. The surfaces were magnified at 1,000X in a scanning electron microscope (Bruker).

Next, a chemical analysis (EDS) was performed using the same samples that were submitted to SEM to describe the chemical composition present on the ceramic surface after each surface treatment. The EDS spectrometer works coupled to the SEM using the Bruker system with the Bruker software program at an accelerating voltage of 20kV and a working distance of 17.5 mm.

2.5 Statistical analysis

The study power was calculated using the OpenEpi website (www.openepi.com), considering a 95% confidence interval and 15 samples per experimental group. The Shapiro-Wilk test was performed to assess normality.

The data obtained in the shear strength test were submitted to the 2-way analysis of variance (ANOVA) and the Tukey’s Test (5%) to compare the experimental groups with each other using the STATISTIX computer program (Analytical Software Inc., version 8.0, 2003).

In addition, the Weibull analysis was performed for a more precise description of the reliability of the ceramic material and strength variation. The Weibull modulus and characteristic strength, with a 95% confidence interval, are determined from the equation:


$$lnln\left(\frac{1}{1-F\left(\sigma_{c}\right)}\right)=mln\sigma_{c}-\;mln\sigma_{0}$$ (1)

In which: F represents the probability of failure; σ_0_ is the initial strength; σc is the characteristic strength; and m is the Weibull modulus. The characteristic strength is considered to be the strength at which the probability of failure is approximately 63%.

## Results

The power of the sample was 100%, considering the two-tailed 95% confidence interval. The results of the Shapiro-Wilk test indicated normal and homogeneous data distribution (P > 0.05).

### Shear bond strength

ANOVA (2-factor) revealed that the “surface treatment” (p=0.0000) and “storage” (p = 0.0000) factors were statistically significant, but the interaction between them did not significantly influence the shear bond strength (Table 1). Tukey’s test revealed that the treatment with silicatization (Si_ST_: 30.47^A^ MPa/Si: 29.21^A^ MPa) resulted in statistically higher bond strength values than the other groups, regardless of storage. Higher values for the C groups were recorded for the C group (16.12^C^ MPa) compared to the C_ST_ group (10.80^D^ MPa). The groups submitted to blasting with aluminum oxide also showed high bond strength values (Al: 21.89^B^ MPa), with only the Al_ST_ group (19.19^BC^ MPa) being statistically similar to the C group (16.12^C^ MPa). The groups treated with Zirlink (Z_ST_: 2.76^F^ Mpa/Z 5.27^EF^ MPa) showed the lowest shear strength values, only similar to the Gl_ST_ group (5.14^EF^ MPa) (Table 2).

**Figure 2:**
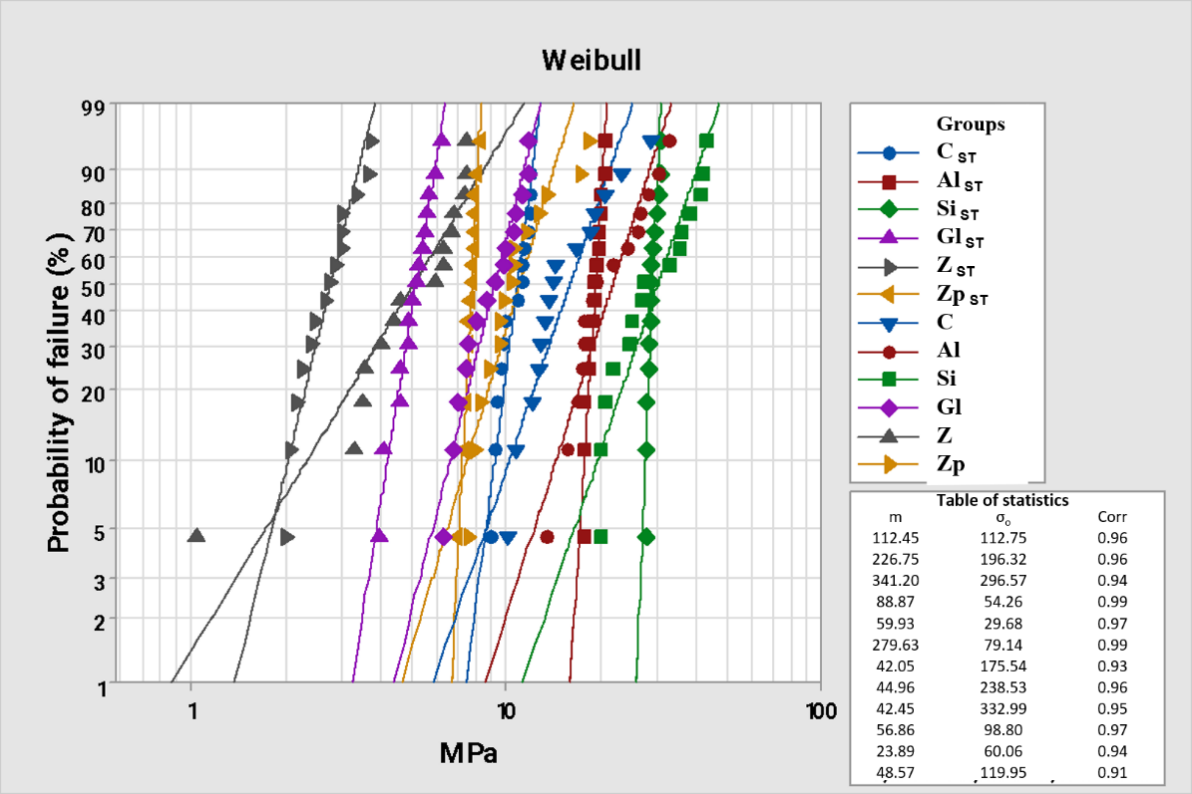
Weibull plot for shear strength. C_ST_ - control with storage; Al_ST_ - sandblasting with Al_2_O_3_ with storage; Si - silicate with storage; Gl_ST_ - glaze with storage; Z_ST_ - zirlink with storage; Zp_ST_ - zirlink + primer with storage; C - control without storage; Al - sandblasting with Al_2_O_3_ without storage; Si - silicate without storage; Gl - glaze without storage; Z - zirlink without storage; Zp - zirlink + primer without storage.

The Weibull modulus (m) revealed a statistical difference between the groups (p = 0.000). The characteristic Weibull resistance was statistically significant (p < 0.0001) and was within expectations about shear bond strength values (Tukey’s test), consequently indicating low variability and greater reliability. The Si group obtained higher values than the others, being statistically similar to the Si^ST^ group, the Zirlink treatment was proven to have the lowest value for characteristic resistance, being statistically similar to the Gl_ST_ group. The Weibull analysis, including the characteristic strength (σ_0_) and the Weibull modulus (m) and their statistical differences are described in Table 2 and Figure 2.

**Table 1:** Two-way ANOVA results for shear strength data.

Source	DF	SS	MS	F	P
Treatment	5	13841.0	2768.20	211.78	0.0000*
Storage	1	457.9	457.89	35.03	0.0000*
Treatment*Storage	5	72.4	14.47	1.11	0.3585
Error	168	2196.0	13.07		
Total	179	16567.2			

DF: degree of freedom; SS: sum of square; MS: mean square; f: F-statistic; ∗∗ Significant statistic (p < 0.05).

**Table 2:** Mean shear strength (MPa) with standard deviation, characteristic strength (σ_o_), Weibull modulus (m), and respective CI (95%) for shear strength of experimental groups.

Groups		Bond strength (MPa)	**Weibull modulus (*m*)**	**95% CI for (*m*)**	Characteristic resistance (σo)	95% CI for (σ_o_) (MPa)
Treatment	Storage					
C	Without	16,12^C^±5,15	4,20^a^	3,23±5,47	17,55^c^	15,38±20,02
Al		21,89^B^±5,93	4,49^a^	3,17±6,35	23,85^bc^	21,14±26,90
Si		30,47^A^±8,58	4,24^a^	2,88±6,25	33,30^a^	29,33±37,79
Gl		9,17^DE^±1,85	5,68^a^	3,73±8,65	9,87^d^	8,99±10,85
Zp		11,10^D^±3,16	4,85^a^	3,78±6,23	11,99^d^	10,68±13,46
Z		5,27^EF^±1,92	2,39^a^	1,20±4,74	6,00^ef^	4,79±7,52
C	With (_ST_)	10,80^D^±1,13	11,24^ac^	7,02±18,01	11,27^d^	10,75±11,82
Al		19,19^BC^±1,01	22,67^bc^	15,52±33,11	19,63^c^	19,17±20,10
Si		29,21^A^±1,05	34,11^b^	25,13±46,31	29,65^ab^	29,18±30,13
Gl		5,14^EF^±0,65	8,88^ac^	5,75±13,72	5,42^f^	5,10±5,76
Zp		7,76^DE^±0,32	27,91^bc^	18,06±43,14	7,91^e^	7,76±8,06
Z		2,76^F^±0,55	5,98^a^	4,21±8,51	2,96^f^	2,71±3,24

Shear Bond Strength. Different case superscript letters indicate significant differences between groups for bond strength. Letters of different superscript suspects indicate a significant difference between groups for the Weibull modulus. C - control; Al - sandblasting with Al_2_O_3_; Si - silicate; Gl - glaze; Z - zirlink; Zp - zirlink + primer.

### Surface roughness

ANOVA (1-factor) revealed that the “surface treatment” (p = 0.0000) factor was statistically significant. Turkey’s test for Ra revealed statistical similarity for all groups. The Z group (0.4049^DE^μm) presented the highest values for Rq, being statistically similar to the Al (0.2516^EFGH^μm) and Si (0.3160^EFG^μm) groups (Table 3).

The Al group (1.7894^A^ μm) showed the highest values for Rz, followed by the Si group (1.5019^B^ μm), which was also statistically different from the others. The C Group (0.3356^EF^ μm) obtained the lowest values, followed by the Gl group (0.5343^D^ μm) (Table 3).

**Table 3:** Mean roughness (Ra); mean square roughness (Rq); and mean roughness in the Z dimension (Rz).

Groups	Ra (μm)	Rq (μm)	Rz (μm)
C	0,0774^H^	0,1199^H^	0,3356^EF^
Al	0,2304^EFGH^	0,2516^EFGH^	1,7894^A^
Si	0,2511^EFGH^	0,3160^EFG^	1,5019^B^
Gl	0,1641^FGH^	0,1104^H^	0,5343^D^
Z	0,2593^EFGH^	0,4049^DE^	1,0528^C^
Zp	0,2059^FGH^	0,1410^GH^	1,0091^C^

C - control; Al - sandblasting with Al_2_O_3_; Si - silicate; Gl - glaze; Z - zirlink; Zp - zirlink + primer.

### Failure analysis

**Figure 3:**
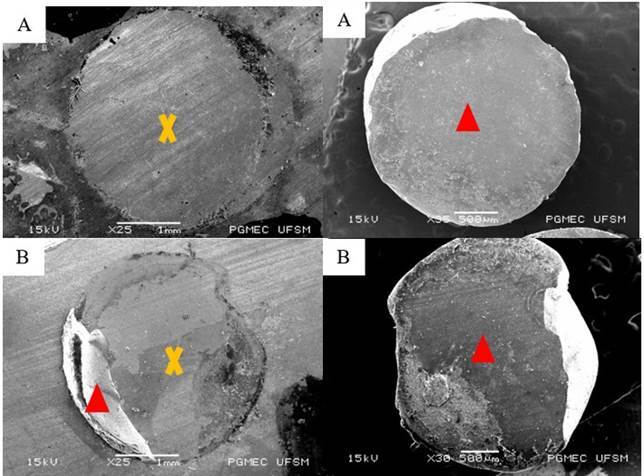
Scanning electron micrographs, representing surface flaws after testing the bond strength between ceramic and resin cement cylinders. A) adhesive - between the ceramic/cement interface; B) mixed 2 - adhesive between the cement/ceramic + cement cohesive interface. ∆ (red): cement; x (yellow): ceramic.

The types of failures found most frequently were adhesive and mixed 2, with the first occurring more frequently in the Gl and Z groups, while the second predominated in the C, Al, Si, and Zp groups. None of the groups had ceramic cohesive type failure. The predominance of the type of failure in each experimental group can be observed in Table 4, and the SEM of representative samples of the failure patterns found can be seen in Figure 3.

**Table 4:** Surface failure analysis of specimens submitted to the shear strength test.

Groups	Types of failures (%)			
	Adhesive	Mixed 1: adhesive + cohesive ceramic interface	Mixed 2: adhesive + cement cohesive interface	Ceramic cohesive
C	7 (46,6)	0 (0)	8 (53,3)	0 (0)
Al	4 (26,6)	0 (0)	11 (73,3)	0 (0)
Si	3 (20)	0 (0)	12 (80)	0 (0)
Gl	12 (80)	0 (0)	3 (20)	0 (0)
Z	10 (66,6)	0 (0)	5 (33,3)	0 (0)
Zp	6 (40)	0 (0)	9 (60)	0 (0)

C - control; Al - sandblasting with Al_2_O_3_; Si - silicate; Gl - glaze; Z - zirlink; Zp - zirlink + primer.

## SEM/EDS

**Figure 4:**
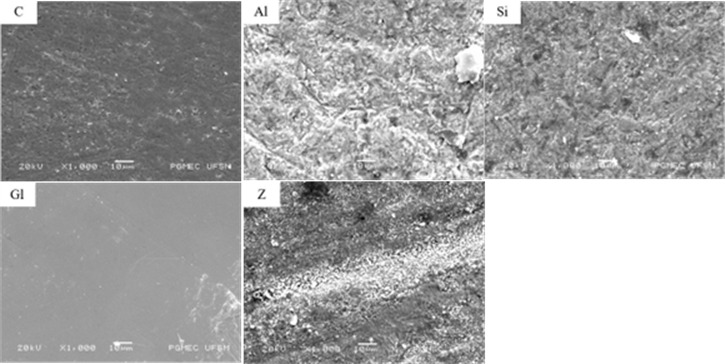
Scanning electron micrographs at ×1000 magnification. Topographic changes on the zirconia surface after each surface treatment: C - control; Al - sandblasting with Al_2_O_3_; Si - silicate; Gl - glaze; Z - zirlink.

The Si and Al groups showed greater topographical change compared to the C group, and it was possible to observe irregularities on the ceramic surface, even more evident in the Al group. The Gl group has a regular surface. It is possible to observe microgranulations in the material in group Z. Despite group C not having received any surface treatment, it has some micropores, which may be the result of ceramic contact with abrasive water sandpaper during the manufacture of ceramic blocks. SEM micrographs are shown in Figure 4.

Regarding the EDS analysis, the amount of silica increased after surface treatments compared to group C, except for group Al. This change was more evident for the Gl group, followed by the Si group. On the other hand, the zirconium and yttrium percentages decreased, mainly in the Gl group (Table 5).

**Table 5:** Mass content (%) of ceramics after each surface treatment by EDS analysis.

Elements	Surface treatment					
	C	Al	Si	Gl	Z	Zp
Oxygen	13,00	19,51	17,74	33,22	14,00	15,26
Silicon	0,00	-	0,83	31,59	0,14	0,15
Carbon	13,46	16,70	21,12	10,44	-	19,16
Potassium	-	-	-	8,64	-	0,00
Aluminum	-	2,63	1,76	6,83	-	-
Zirconium	68,49	56,75	54,43	0,00	63,00	61,05
Calcium	-	-	-	3,51	18,84	-
Titanium	-	-	-	0,32	-	-
Magnesium	-	-	-	0,32	-	-
Yttrium	5,05	4,41	4,12	0,00	4,02	4,39
Sodium	-	-	-	5,13	-	-
Gold	0,00	0,00	0,00	0,00	0,00	0,00

C - control; Al - sandblasting with Al_2_O_3_; Si - silicate; Gl - glaze; Z - zirlink; Zp - zirlink + primer.

## Discussion

Three types of tests are generally used to evaluate the bond strength of the ceramic-resin cement interface, such as microshear, tensile, and microtensile. The test used for this study was the shear test, as the hardness and high strength of zirconia make it very difficult to section the sintered samples to perform the microtensile test ^12^; in addition, many premature failures are induced at the interface during this process, decreasing the reliability of the test ^12^.

The hypothesis that Zirlink has higher shear bond strength values compared to other surface treatment methods for ultratranslucent zirconia was rejected. The results showed that the group treated with silicatization obtained superior shear strength values to the others; while the strength of these groups after treatments with Al, Zp, and Gl was similar to group C, in which only Primer was applied to zirconia. These results corroborate the findings of Ozcan, Cura, and Valandro (2011) ^13^, in which superior bond strength results were observed for the silicatization process associated with silanization when compared to aluminum oxide blasting associated with silanization. This behavior is due to the chemical bond between the aluminum oxides and the silane, which presents greater potential for hydrolytic degradation than between the silica-coated aluminum oxide and the silane ^8^.

Furthermore, according to Souza, Ozcan, and Miyashita (2012) ^14^, the silica coating is less aggressive than blasting with aluminum oxide for restoration margins, as it uses fewer particles and air pressure. This was verified by the EDS and SEM results, which respectively showed a higher proportion of silica after blasting with SiO2 and less surface aggression compared to the group blasted with aluminum oxide, which visually presents a rougher surface. Kumar et al. (2023) ^6^ revealed that applying mechanical surface treatments on zirconia, such as sandblasting, can promote the transformation from tetragonal to monoclinic phase (a process which is known as transformation toughening), which promotes the formation of a residual layer of compressive stress. Thus, depending on the size and type of the particle and the blasting pressure, these treatments can cause damage to the material’s surface, minimizing the protective effect of the residual compressive layer, or even reducing the ceramic strength, thus negatively impacting the mechanical properties of this material ^15^.

Regarding the new surface treatment with Zirlink, according to the manufacturer, it is capable of promoting adhesion on zirconia with up to seven times more strength than common abrasion techniques using air particles. According to Yong-Bum et al. (2020) ^16^, the application of a ZrO_2_ suspension similar to Zirlink, with carbon nanoparticles onto the surface of pre-sintered zirconia resulted in microstructural changes in the ceramic through increased porosity and surface roughness, improving its bond strength to resinous cement. An increase in surface roughness was also observed in this study after treatment with Zirlink, as well as microgranulations on the ceramic surface being confirmed by SEM analysis. However, these microstructural changes, which should cause mechanical interlocking at the adhesive interface ^17^, seem to not be sufficient to increase the zirconia adhesion to the resin cement, since the Z and Zp groups obtained lower shear strength values than the groups submitted to traditional blasting techniques. Corroborating these results, Ji-Hyeon et al. (2021) and Gang-Ho et al. (2021) ^17^ ^,^ ^18^ also obtained higher shear strength values for the groups submitted to blasting when comparing blasting and ZrO_2_ suspension as surface treatments for zirconia.

Furthermore, according to the manufacturer, the increase in bond strength after treatment with Zirlink is due to the presence of nanoceramic components compatible with yttria in the solution. However, this was not observed in the EDS test; on the contrary, the percentage of yttrium decreased after treatment with Zirlink. Colombino et al. (2022) ^19^, when evaluating the effect of surface treatment by ZrO2 suspension for ultratranslucent zirconia, did not obtain good results in bond strength and suggest that the concentration of the ZrO2 suspension may be an important factor in changing the surface microstructure. Furthermore, although the Z and Zp groups received the same surface treatment, higher bond strength values for the Zp group can be attributed to the use of Primer for zirconia, which acted as an adhesion agent for ceramics to resin cement. This is due to the fact that this primer is an alcoholic solution of silane methacrylate, sulfide methacrylate, and phosphoric acid methacrylate, which in turn has MDP that chemically reacts with zirconia dioxide, promoting a stable union to zirconia. Furthermore, applying primer to the ceramic surface increases the surface energy by increasing wettability, which favors cement flow and chemical interaction between resin cement and zirconia. However, Pott, Stiesch, and Eisenburgerthis (2015) ^20^ confirm that durable bonding to zirconia ceramics cannot be achieved with MDP-containing cement without surface treatment, this chemical bond may be sensitive to hydrolysis, which may result in decreased bond strength under intraoral conditions. Similarly, when evaluating the control group in this study in which only the Primer was applied, lower shear strength values were obtained after storage for 150 days; unlike the group treated with silicatization, which maintained high bond strength values even after storage.

The second hypothesis that different surface treatment protocols affect the surface roughness of ultratranslucent zirconia was accepted. We measured Rq (root mean square roughness) and Rz (maximum height of the roughness profile based on peaks and valleys) in addition to Ra in our study to increase the accuracy of the test. Thus, when the Ra values are similar, as occurred herein, we can base the Rq and Rz values on surface roughness. The surface treatments performed on the restoration must promote roughness to have strong bonding at the zirconia-resin cement interface, as well as to chemically activate it so that it can imbricate and adhere to the cement, respectively ^8^. So, although the Al group obtained the highest Rz values, which justifies higher shear strength values for the Si group is the sum of the mechanical bond with a chemical bond obtained in the silicatization process.

Regarding the failure analysis, the predominance of mixed cohesive cement failures is related to higher adhesion values between zirconia and resin cement ^3^. This corroborates the data obtained in this study, since the Si group had the highest percentage of mixed failures and the highest shear strength values, while adhesive-type failures predominated for the Gl and Z groups. 

Future research is needed to evaluate the adhesive behavior of the surface treatment method using Zirlink for ultratranslucent zirconia, as well as the use of other types of cement and adhesive systems. A limitation of this study is that only one type of zirconia was used; exploring other storage methods, such as thermocycling, could provide additional insights. Finally, randomized controlled clinical studies are also needed to validate the results found in this in vitro study.

## Conclusion

Based on the results obtained, it can be concluded that silicatization associated with silanization is the most suitable surface treatment method for ultratranslucent zirconia, to improve the bond strength to the resin cement. The application of Zirlink and Glaze on pre-sintered zirconia did not promote efficient adhesion of the ultratranslucent zirconia to the resin cement, even when associated with a primer containing MDP.
